# *Dracunculus* sp. PantanalBr Infection in Florida Panthers and Bobcat, Florida, USA

**DOI:** 10.3201/eid3207.260514

**Published:** 2026-07

**Authors:** Michael J. Yabsley, Alexander Perez, Kayla B. Garrett, Christopher A. Cleveland, Mark Cunningham, Peter Sebastian, Bambi Clemons, Jeff M. Gruntmeir, Heather D.S. Walden

**Affiliations:** Warnell School of Forestry and Natural Resources and Southeastern Cooperative Wildlife Disease Study, University of Georgia, Athens, Georgia, USA (M.J. Yabsley); University of Georgia, Athens (M.J. Yabsley, K.B. Garrett, C.A. Cleveland); University of Florida, Gainesville, Florida, USA (A. Perez, J.M. Gruntmeir, H.D.S. Walden); Florida Fish and Wildlife Commission, Fish and Wildlife Research Institute, Gainesville (M. Cunningham, P. Sebastian, B. Clemons)

**Keywords:** *Dracunculus* sp. PantanalBr, parasites, worms, Florida panthers, bobcat, Florida

## Abstract

We used morphologic and genetic methods to analyze subcutaneous worms removed from endangered Florida panthers and a bobcat in Florida, USA, identifying *Dracunculus* sp. PantanalBr and several *Dirofilaria* spp. worms. *Dracunculus* sp. PantanalBr had been previously reported in a domestic dog and a jaguar in Brazil.

*Dracunculus* (Spirurida:Dracunculoidea) are large subcutaneous nematodes that can be found in mammals and reptiles (*1*). The life cycle of the parasites involves ingestion of infected cyclopoid copepods via drinking water, although consumption of paratenic or transport hosts (amphibians, fish) may also be involved (*1**,**2*). Female *Dracunculus* nematodes are morphologically indistinguishable by species and more commonly detected than the much smaller male nematodes, so sequence analysis is necessary for species identification (*1*).

Six of 15 *Dracunculus* species infect mammals, and most studies focus on the human Guinea worm, *Dracunculus medinensis*, in Africa (*1*). In North America, studies have reported 4 mammalian *Dracunculus* spp. nematodes: *D. insignis* (in various wild carnivores, dogs, cats), *D. lutrae* and an undescribed species (in river otters [*Lontra canadensis*]), and another undescribed species (in a Virginia opossum [*Didephis virginianus*], a river otter [USA], and a dog [Spain]) (*1*,*3*–*5*). Researchers have reported 3 *Dracunculus* nematode species in South America: *D. jaguape* (in a neotropical otter [*Lontra longicaudis*]), *D. fuelleborni* (in a big-eared opossum [*Didelphis aurita*]), and an undescribed species (*Dracunculus* sp. PantanalBr) (in dogs and a jaguar [*Panthera onca*] in Brazil) (*6*,*7*).

The Florida panther (*Puma concolor coryi*) is an endangered North American puma (*P. c. couguar*) subspecies restricted to South Florida. As part of mortality investigations, Florida Fish and Wildlife Conservation Commission veterinarians perform necropsies on panthers and bobcats (*Lynx rufus*). This study reports the findings related to worms collected from anthers and a bobcat, including detection of *Dracunculus* sp. PantanalBr.

We removed subcutaneous or internal parasites from 12 Florida panthers and 1 bobcat collected in Florida during 2002–2025, preserving the samples in formalin or 70% ethanol (Figure 1, panel A; Appendix 1 Table). We morphologically identified and genetically characterized all worms collected (Appendix 1).

**Figure 1:**
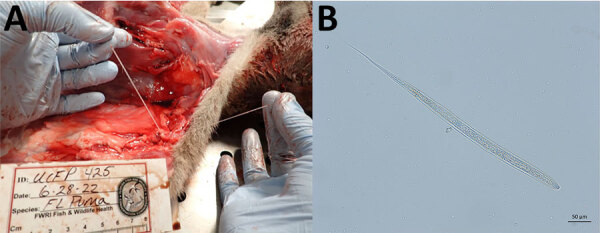
*Dracunculus* sp. PantanalBR nematode samples collected in investigation of *Dracunculus* sp. PantanalBr infection in Florida panthers and a bobcat, Florida, USA. A) Female *Dracunculus* sp. PantanalBR nematode was detected in subcutaneous tissues of a Florida panther (*Puma concolor coryi*). B) First-stage larvae of female *Dracunculus* sp. PantanalBR from a Florida panther. Original magnification ×200.

Most worms were fragments, so we based identification on a combination of sequence analysis and morphology (characteristic first-stage larvae) (Figure 1, panel B). We noted 5 panthers and the bobcat to be infected with *Dracunculus* sp. nematodes, subsequently identifying parasites from 2 of those panthers and the bobcat as *Dracunculus* sp. PantanalBR. We identified *Dirofilaria* spp. nematodes in 7 panthers (Appendix 1).

We obtained partial *Dracunculus* cytochrome oxidase subunit I (COI) and 18S rRNA sequences from 2 Florida panthers and the bobcat. The two 657-bp COI sequences from Florida panthers were identical and were 99.7% similar to *Dracunculus* sp. PantanalBR identified in a jaguar and 98.8% similar to *Dracunculus* sp. PantanalBR detected in a dog (Appendix 2 Table 1). The bobcat worm sequence was 99.5% (654/657 bp) similar to the Florida panther sequences. Phylogenetically, the Florida panther and bobcat worm sequences grouped with the 2 *Dracunculus* sp. PantanalBR sequences (Figure 2, panel A). The 18S rRNA sequences (956 base pair) from the 2 Florida panthers and bobcat were identical and 99.9% similar to *Dracunculus* sp. PantanalBR (806/807 bp) (Appendix 2 Table 2). Phylogenetic analysis produced a similar tree to the COI gene (Figure 2, panel B). Larvae from *Dracunculus* sp. PantanalBR from 1 panther measured 601.67 µm long and 25.56 µm wide.

**Figure 2:**
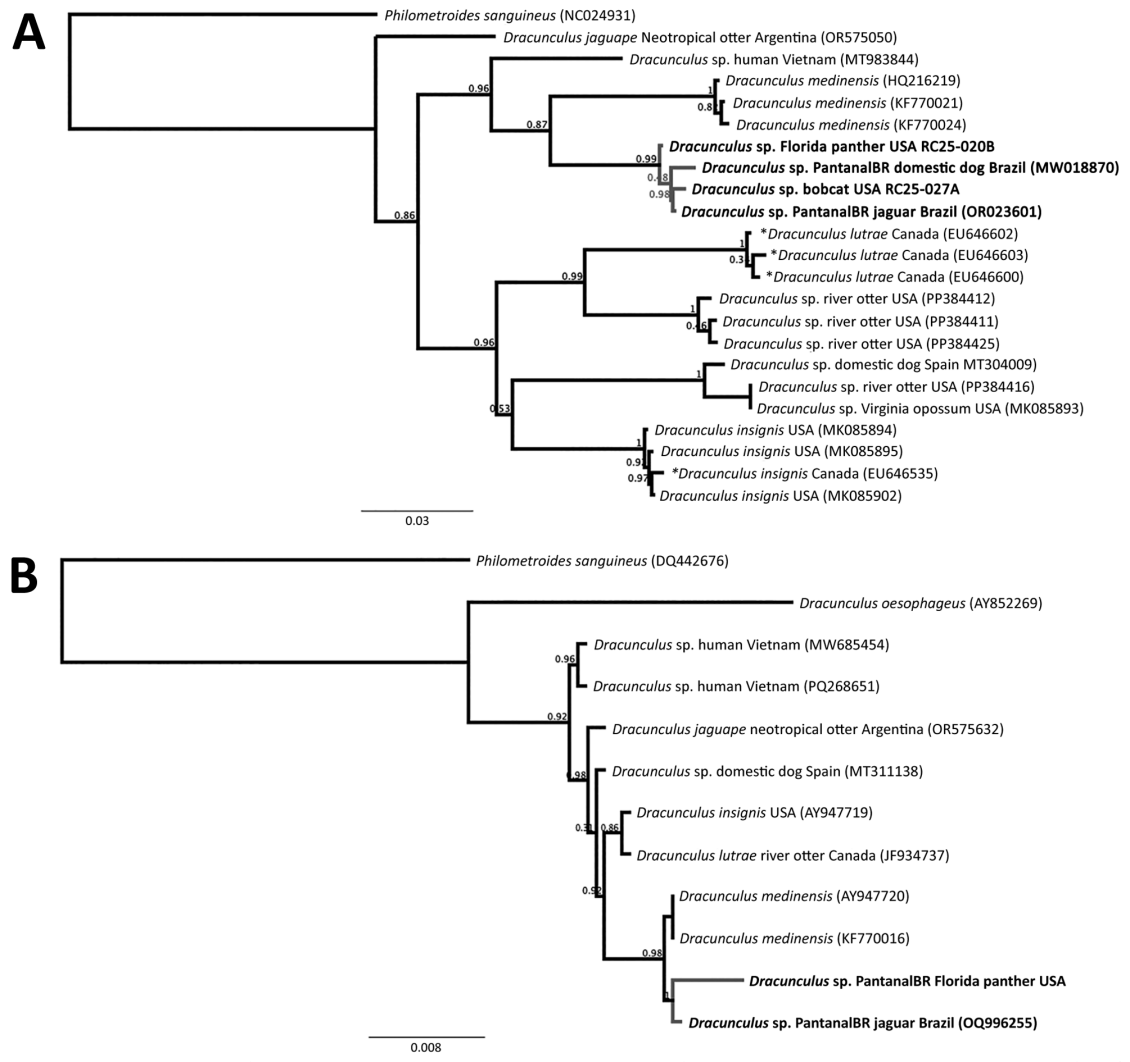
Phylogenetic tree of *Dracunculus* sp. PantanalBr nematodes collected from Florida panthers and a bobcat, Florida, USA. A) Genetic relationships of *Dracunculus* sp. PantanalBR from a Florida panther (*Puma concolor coryi*) and a bobcat (*Lynx rufus*) compared with other *Dracunculus* spp. based on partial cytochrome c oxidase subunit 1 gene sequences. B) Genetic relationships of *Dracunculus* sp. PantanalBR from Florida panther compared with other *Dracunculus* spp. based on partial 18S rRNA gene sequences. Boldface text represents specimens analyzed in this study. Scale bars indicate substitutions per site.

Our data confirm *Dracunculus* sp. PantanalBR nematodes in North America. A prior report of female *Dracunculus* nematodes in Florida panthers was reported as *D. insignis* in 2 Florida panthers from Monroe County in 1989–1990; however, that investigation included no genetic analysis to confirm species (*8*). Thus, it is unknown if Florida panthers are hosts for *Dracunculus* sp. PantanalBR and *D. insignis* nematodes, although *D. insignis* nematode infects domestic cats (*5*). Before our report, researchers reported 3 *Dracunculus* nematode species in Florida, including 2 undescribed *Dracunculus* clades in 2 river otters and unspeciated female *Dracunculus* nematodes in a domestic dog and raccoons (*1**,**3**,**4*). However, because *Dracunculus* sp. PantanalBR infects dogs and *D. insignis* nematode is only presumed to occur in Florida, worms from dogs and cats should be genetically characterized to determine species.

The *Dracunculus* sp. PantanalBR life cycle is unknown, but *Dracunculus* nematode species use copepods as intermediate hosts, and some species may use aquatic paratenic hosts (*1*,*2*). Further studies are needed to determine if this parasitic species is transmitted through ingestion of copepods or through a paratenic host. We discovered the subcutaneous parasites in the animals we studied during routine necropsy, and no lesions were noted, but researchers have observed ulceration and edema in other *Dracunculus*-infected hosts (*1**,**3**–**7*). *Dracunculus* infections can cause lameness in some hosts, but observation of clinical signs in free-ranging wildlife might be difficult.

The Florida panther is restricted to southern Florida, and the source of *Dracunculus* nematodes in the population is unknown. Genetic testing of panthers in Florida identified a unique lineage in the Everglades National Park that appeared to be of South American origin (*9*,*10*). Researchers presumed the origin of this unique genotype was the introduction of 7 captive pumas in the 1950–1960s to the park, all of which were brought from Central America (*9*,*10*). Although that history suggests a possible introduction route for *Dracunculus* sp. PantanalBR nematodes, additional surveillance of canids and felids in the Americas is needed to further clarify distribution and risk for infection.

Appendix 1 Methods and species data for *Dracunculus* sp. PantanalBr infection in Florida panthers and bobcat, Florida, USA.

Appendix 2 Additional information for *Dracunculus* sp. PantanalBr infection in Florida panthers and bobcat, Florida, USA.
