# Supplementary material for: Dracunculus sp. PantanalBr Infection in Florida Panthers and Bobcat, Florida, USA
Source: Emerg Infect Dis. 2026 Jul;32(7):1214–7. doi: 10.3201/eid3207.260514 (PMC13322447; doi:10.3201/eid3207.260514)
Supplement: Appendix 1 — Methods and species data for Dracunculus sp. PantanalBr infection in Florida panthers and bobcat, Florida, USA. [file 26-0514-Techapp-s1.pdf]

Article DOI: http://doi.org/10.3201/eid3207.260514

EID cannot ensure accessibility for supplementary materials supplied by authors. Readers who have difficulty accessing supplementary content should contact the authors for assistance.

Dracunculus sp. PantanalBr Infection in Florida Panthers and Bobcat, Florida, USA

Appendix 1

Methods

Molecular methods

Genomic DNA was extracted from the nematodes using a QIAGEN DNeasy Blood and Tissue Extraction Kit (Germantown, MD, USA) following manufacturer's directions. Portions of the 18S rRNA (950bp) and cytochrome c oxidase I (COI) (650bp) genes were amplified using primers 18S39F and 18S977R (*1*) and a cocktail of three primers, respectively (*2*). Amplicons were visualized in a 1% agarose gel stained with GelRed (Biotium, Fremont, CA, USA) and then extracted from the gel using a QIAGEN gel extraction kit per manufacturer's directions. Bi-directional Sanger sequencing was conducted by Genewiz (South Plainfield, NJ, USA). Sequences were edited, primer sequences were removed, and assembled using Geneious 10.2.6 (Biomatters Limited, Auckland, New Zealand) and aligned with related sequences from GenBank. Phylogenetic trees were constructed in Geneious using an approximately maximum-likelihood method with FastTree v2.1 with a generalized time-reversible (GTR) model. Unique sequences from the Florida panther worms were submitted to Genbank (accession numbers PZ530940, PZ530945, and PZ530946).

The use of materials collected from wildlife during routine necropsies by state wildlife veterinarians for diagnostics has been reviewed and approved by the University of Georgia’s Institutional Animal Care and Use Committee (A2024 02–014).

Results and Discussion

Seven of the 16 panthers with worms were infected with *Dirofilaria* spp., including four with *Dirofilaria striata*, two with *Dirofilaria immitis,* and one with a *Dirofilaria* sp. (this partial sample was in formalin and could not be genetically identified to species). Partial 18S rRNA

(822bp) gene sequences from two *Dirofilaria* specimens were identical and 100% similar to *D. striata* (MN635455) from a domestic cat from Florida (*3*). The partial COI (497) was 99.6% similar to *D. striata* from domestic cats from Florida (MN635457) and Texas (PX315770). Two additional *Dirofilaria* samples were morphologically identified as *D. striata* based on morphology of male worms. Finally, worms from two panthers were identified as *D. immitis*; one was based on morphology of adult worms collected in the heart and the other by analysis of COI sequences that were 100% identical to numerous sequences in GenBank. *Dirofilaria striata* has been previously reported from Florida panthers, bobcats, and domestic cats in Florida and it can cause subcutaneous nodules in domestic cats (*3*,*4*). *Dirofilaria immitis*, found in two panthers, is common in dogs in Florida and can cause disease in felids (www.capcvet.org).

References

1. Olson M, Harris T, Higgins R, Mullin P, Powers K, Olson S, et al. Species delimitation and description of *Mesocriconema nebraskense* n. sp. (Nematoda: Criconematidae), a morphologically cryptic, parthenogenetic species from North American Grasslands. J Nematol. 2017;49:42–66. PubMed https://doi.org/10.21307/jofnem-2017-045

2. Prosser SW, Velarde-Aguilar MG, León-Règagnon V, Hebert PD. Advancing nematode barcoding: a primer cocktail for the cytochrome c oxidase subunit I gene from vertebrate parasitic nematodes. Mol Ecol Resour. 2013;13:1108–15. PubMed https://doi.org/10.1111/1755-0998.12082

3. Wyatt D, Santoro D, Deabold K, Gruntmeir J, Childress A, Craft WF, et al. Subcutaneous nodules and dermatitis associated with non-*immitis* non-*repens* dirofilariosis morphologically consistent with *Dirofilaria striata* in a 2-year-old male domestic cat in Florida, USA. Vet Q. 2020;40:215–22. PubMed https://doi.org/10.1080/01652176.2020.1814972

4. Forrester DJ. Parasites and Diseases of Wild Mammals in Florida. 1992. Gainesville, FL: University Press of Florida; 459 pp.

Appendix 1 Table. Summary data for *Drancunculus* and *Dirofilaria* spp. from Florida panthers (*Puma concolor coryi*) and a bobcat (*Lynx rufus*) in Florida, USA.

Species	Date	County	Sex	Age, y	Site of parasite	Parasite ID based on morphology	Parasite ID based on sequencing
Florida panther	5/14/22	Lee	F	10mo	subcutaneous	*Dracunculus* sp.	*Dracunculus* sp. PantanalBR
Florida panther	7/3/24	Collier	M	2	subcutaneous	*Dracunculus* sp.	*Dracunculus* sp. PantanalBR
Florida panther	4/12/21	Lee	F	1.5	subcutaneous	*Dracunculus* sp.	ND
Florida panther	5/21/19	Lee	M	1.8	abdomen	*Dracunculus* sp.	ND
Florida panther	4/30/11	Collier	M	1.5–2	subcutaneous	*Dracunculus* sp.	ND
Florida panther	1/9/22	Glades	M	3	subcutaneous	*Dirofilaria striata*	*Dirofilaria striata*
Florida panther	2/23/25	Hendry	F	10.5	xyphoid	*Dirofilaria striata*	*Dirofilaria striata*
Florida panther	7/10/11	Collier	M	1.5	subcutaneous	*Dirofilaria striata*	ND
Florida panther	3/25/11	Collier	F	11y, 10mo	subcutaneous	*Dirofilaria striata*	ND
Florida panther	7/5/11	Collier	M	8	subcutaneous	*Dirofilaria* sp.	ND
Florida panther	3/25/21	Collier	F	7	heart	*Dirofilaria immitis*	*Dirofilaria immitis*
Florida panther	4/5/02	Glades	M	3	heart right ventricle	*Dirofilaria immitis*	ND
Bobcat	7/11/18	Collier	M	1.5	subcutaneous	*Dracunculus* sp.	*Dracunculus* sp. PantanalBR
